# Experts’ boundary work on social media: Doctors on Instagram

**DOI:** 10.1177/09636625261450028

**Published:** 2026-06-03

**Authors:** Elina Uutela, Essi Pöyry, Salla-Maaria Laaksonen, Mikko Jauho

**Affiliations:** 1 Faculty of Social Sciences, University of Helsinki, Finland; 2 Centre for Consumer Society Research, Faculty of Social Sciences, University of Helsinki, Finland

**Keywords:** boundary work, doctors, expansion of expertise, Instagram, social media

## Abstract

Science-based experts have started to utilize social media to seek direct contact with audiences and social recognition for their expertise. Experts need to balance professional principles and the expectations arising from the changing media landscape. To understand how science-based experts navigate their way on social media, we utilize the concept of boundary work. We analyze how medical doctors enact and expand expertise on Instagram via boundary work in relation to publics, self, and markets. Using qualitative content analysis, we studied 2125 Instagram posts from the 20 most-followed doctors in Finland. Doctors enacted expertise by holding on to their epistemic authority, maintaining their professional role at the core of their posts, and avoiding advertising medical services. However, they expanded expertise by building relationships with their audiences, using anecdotes and personal experiences in their communication, and creating new communicational products for their audiences.

## 1. Introduction

In this article, we study how medical doctors uphold and renegotiate the boundaries of their expertise when they enter the social media platform Instagram.

Social media operates with less gatekeeping than legacy media and values engagement and attention over news value and fact-checking. It has provided an unprecedented space for the dissemination of unverified health information, but also new avenues to engage with various publics. The changing media landscape has given rise to new public actors with claims to expertise, including self-taught field experts (Setälä and Väliverronen, 2014) and lifestyle experts (Lewis, 2010), patient advocates (Egher, 2020; Shanahan, 2010), and social media influencers (Leader et al., 2021; Pöyry et al., 2022), who use social media to engage with audiences, recruit followers, and discuss health topics. These actors sometimes challenge and even contradict scientific consensus, but also often conform to the views of epistemic authorities and supplement existing knowledge with their own experiences.

However, also traditional experts such as medical doctors have entered social media. Previous research has highlighted how medical doctors use social media for public health communication (Furstrand et al., 2021; Pöyry et al., 2022), debunking medical misinformation (Bautista et al., 2021; Mourali and Drake, 2022), and interacting with patients (Brown et al., 2014; Wu et al., 2018), as a complementary way of providing medical education (Katz and Nandi, 2021), or to promote their businesses (Atef et al., 2023; Joa and Park, 2021).

This study introduces a novel perspective into research on medical doctors’ social media use. We approach doctors as traditional science-based experts, who need to renegotiate their expert role when engaging with novel publics in social media. Thus, we approach doctors’ social media use from the perspective of the expansion of expertise (Collins and Evans, 2002; Rask, 2008; Saikkonen, 2019). This concept typically refers to situations where expert status is claimed by new actors without conventional scientific or vocational training that often operate in social media. We apply this concept to the opposite situation where experts with such training expand the definition of expertise when entering social media.

Using platforms like Instagram can make medical professionals and scientific knowledge easier to approach, offering audiences new ways to relate to experts and science. However, previous research indicates that experts who shared personal details were considered less trustworthy, as it feels “out of place” for scientific experts (Zhang and Lu, 2023). Doctors and other traditional experts thus face a balancing act between being visible on new platforms while maintaining their credibility and status. As doctors use social media platforms and adopt new communication tactics, they might also need to develop strategies to set themselves apart from misinformation and the new public experts.

In this study, we analyze how medical doctors enact and expand their expertise through communication on social media. We discuss this as a socially negotiated demarcation between expertise and non-expertise, that is, as boundary work, a concept originally used by Thomas Gieryn (1983) to analyze the distinction between science and other intellectual activities. Building on both theoretical premises and our empirical insights, we differentiate between three dimensions of this boundary work in our case: in relation to publics, the self, and markets.

Our data consist of the top 20 most-followed doctors’ Instagram posts in Finland from 2020 to 2021 (*n* = 2125). This time period was marked by the COVID-19 pandemic, which prompted many medical experts to engage more actively on social media. Although the posts seldom address expertise directly, they position their authors in ways that draw boundaries regarding how professionals should engage with their audience, share aspects of their personal lives, and market products or services.

In what follows, we first explain how the expansion of expertise and related societal developments create pressure for experts’ boundary work on social media. We note three such developments: the public challenging of traditional epistemic authorities, the personified tactics used by novel self-claimed experts, and the rise of promotional culture. Second, we introduce our data and the analytical process. Third, we introduce our findings via the types of boundary work that doctors do on Instagram. Finally, we conclude with a summary, reflect on the study’s limitations, and suggest topics for future research.

## 2. Expertise and its expansion

Expertise is traditionally defined by formal education, scientific knowledge, and professional organization (Collins and Evans, 2002; Grundmann, 2017; Turner, 2001). Medical doctors are a classic example of traditional experts: licensed professionals with a formal education and a code of norms. Another definition of expertise emphasizes the role of experts in society, highlighting their specialized skills and knowledge in solving problems and providing recommendations (Grundmann, 2017; Nowotny, 2000). Doctors fit into this definition, too, as they apply medical science in their work to diagnose and treat patients. In addition, experts also have public roles as trusted advisors. For example, doctors have been involved in organizing the healthcare system and are essential sources of public information on health and illness.

The position of experts and the definition of expertise in society are constantly changing, and their definitions are subject to debate (Eyal, 2019). In science and technology studies, one specific topic of interest has been the broadening participation of various social groups in scientific knowledge production and decision-making. This is called the “extension” (Collins and Evans, 2002) or “expansion” (Rask, 2008) of expertise. Mikko Rask (2008: 23) states that the expansion of expertise refers to “the widening of the actor basis of technical decision-making beyond the core of certified experts through the involvement of non-experts and experience-based experts.” This extension thus covers laypeople or non-experts, as well as new types of experts now regarded as possessing specific lay or experiential expertise.

The expansion of expertise has many drivers and contexts. One is the increasing involvement of citizens in science and technological development. In the medical domain, patient activism and health social movements have shaped care arrangements and even directed research (Hess, 2004; Rabeharisoa and Callon, 2002). Established institutions have involved citizens in decision-making through mechanisms of participatory governance to increase both the knowledge base of decision-making and the legitimacy of the resulting decisions (Rask, 2008). Citizens present relevant, experience-based knowledge of a particular field or subject matter (Demszky and Nassehi, 2012). Such knowledge founds a specific type of expertise, introducing novel actors into the established pool of experts, such as experts by experience (Jones and Pietilä, 2020) and lay experts (Epstein, 1996; Prior, 2003).

In another context relevant to our case, new actors with claims for expertise do not aim to participate in existing knowledge-shaping structures but instead use the changing media environment to address audiences directly. This context is more market-driven: individual citizens’ growing demand for expertise draws new actors to this potential (Grundmann, 2017). While the epistemic authority of established experts is still widely recognized, self-claimed experts get recognition for their insights from their clients and other limited audiences (Turner, 2001; Uutela and Väliverronen, 2025). Consequently, novel forms of public expertise have emerged, often competing or conflicting with traditional experts (Huovila and Saikkonen, 2016; Saikkonen, 2019). They include lifestyle experts (Christensen, 2016; Lewis, 2010), who present desirable lifestyles combined with practical advice, and field experts (Grundmann, 2017; Setälä and Väliverronen, 2014) whose claims to expertise rest on self-learning, accumulated experience of a subject, and a public role in the media. As regards specifically social media, new types of actors with expert claims include online expert mediators, who combine personal experiences with self-taught medical information (Egher, 2020), and social media influencers, who have established a significant number of relevant relationships on social media and are regarded as epistemic authorities by their followers (Enke and Borchers, 2019).

Audiences, then, are navigating through a vastly different media environment than before. Public expertise is more accessible than before, but it is also swamped by noise. There is totally fabricated information and fake news, but also more blurred, misleading information, like facts mixed with false connections, framing, or context (Wardle and Derakhshan, 2017). On the other hand, many trustworthy and established epistemic institutions have built strong social media presences.

In previous research, the expansion of expertise has been approached from the perspective of novel actors, as the proliferation of new types of experts in the new media environment. Our study turns the tables and examines traditional, established experts entering the new media space, renegotiating their expert position within it. We claim that this process has effects that can be seen as an expansion of expertise as professionals accommodate traits associated with experiential experts.

### Boundary work as a negotiation over expanding expertise

We mobilize the concept of boundary work to analyze how medical doctors engage with Instagram as medical professionals. Thomas Gieryn (1983: 781–782) defined boundary work as “the attribution of selected characteristics to the institution of science to construct a social boundary that distinguishes some intellectual activities as ‘non-science.’” The aim of boundary work is to strengthen, redraw, or blur boundaries between clusters or individual actors (Gieryn, 1983). During the over four decades the concept has been in use, boundary work has extended from demarcation of science and non-science to drawing borders between, for example, different professions, roles, and areas of expertise, and trustworthy and untrustworthy knowledge (e.g. Bucher et al., 2016; Carlson and Lewis, 2019; Kjærulff et al., 2024). Erwin Van Rijswoud (2012: 22) analyzes boundary work as something that happens in the “boundary zone between science, politics and the public” and, thus, recognizes it as something scientists do as public experts. In our research, we look at doctors as experts in the public, focusing on how their posts draw the boundaries between professional expertise based on science and professional experience, and other activities.

The concept of boundary work remains widely used to this day, for example, in recent discussions regarding the pressure that generative AI has on professions and their boundaries (Faulconbridge et al., 2025; Van Dalen, 2024). Matt Carlson and Seth Lewis (2019: 125) have summarized the concept’s timeliness—or even timelessness—in their own research about boundary work in journalism through its anti-essentialism:

Boundary work is not a patrol mechanism for some intrinsic scientific value; it is the mechanism through which good science is defined. This view jettisons the search for deeper truth to instead emphasize the surface battles that determine who gets to say what is true. In this way, Gieryn’s boundary work concept is as timely as ever as science faces political contestation around issues such as climate change, vaccination, and genetically modified organisms.

Three developments linked to the expansion of expertise create the need for boundary work for traditional experts: the increasing publicity and scrutiny of experts, the personified tactics used by new forms of expertise, and the rise of promotional culture in professional surroundings. Next, we introduce these dimensions and the need for boundary work they create.

First, experts are more in the public eye and must balance their science-based epistemic authority with public expectations. Experts have gained increasing visibility in the legacy media, establishing themselves as public commentators. Journalists routinely use experts as sources to add trustworthiness and factuality to their articles (Albæk, 2011; Albæk et al., 2003; Boyce, 2006), as experts are a low-cost way to access scientific information (Reich and Godler, 2016). During the COVID-19 pandemic, doctors of various specialties were frequent visitors to TV news shows and essential sources for journalists. By making public appearances, experts have paved the way for using the changing media landscape but also exposed themselves and their professions to public assessment and scrutiny. Publics take part in negotiations over expertise online, mobilizing a variety of arguments from personal experience to moral assessments and scientific argumentation (Shanahan, 2010; Uutela and Väliverronen, 2025). Experts are assessed not only as epistemic authorities but also as service providers, policy commentators, and moral actors. On social media, debates challenging experts and scientific institutions are common. In health, examples range from vaccination (Kata, 2012) to epidemiology (Väliverronen et al., 2020) and proper diet (Gunnarsson and Elam, 2012; Jauho, 2016).

Second, personal experiences and personification mark the style of novel forms of expertise, such as field experts and lifestyle experts. Therefore, experts entering social media need to balance their professional role with personal exposure. Traditional expertise is characterized by impartiality, detachment, and the separation of personal opinions from factual statements, while the emerging forms of experts explicitly foreground the personal. Although often utilizing and thus maintaining the authority of science (Egher, 2020; Shanahan, 2010), the emerging experts aim to make the facts alive through their personal experiences and increase their credibility through personal healing and growth stories (Huovila and Saikkonen, 2016; Jauho, 2016; Pedersen and Baarts, 2010). The same phenomenon has appeared in other media: for example, it has been argued that television’s audience-centered approach replaced medical professionals with more appealing lifestyle experts (Christensen, 2016). To compete with these actors, doctors must incorporate more private or personalized elements into their professional role. Thus, doctors on social media strike a balance between being knowledgeable, like a doctor, and approachable and popular, like an influencer (Atef et al., 2023).

Third, promotional tactics have become a substantial part of public expertise. Esa Väliverronen (2021) notes that a promotional culture has come to characterize science, where advertising, PR, and marketing affect both the outward reach of scientific institutions and the self-promotion of scientists. Simultaneously, commercial social media platforms have risen, becoming a significant part of the public space. In social media, promotion is integral to its operational parameters, such as sharing and liking (Van Dijck, 2013). Partnerships with commercial actors are an established part of this environment and a significant aspect of influencer revenue (Vrontis et al., 2021). Because of this, traditional experts in social media operate in a commercial environment with promotional intentions while aiming to maintain expert impartiality and follow professional norms.

As described above, societal changes have created the need to renegotiate three overlapping types of boundaries doctors on Instagram work with. First, in relation to publics, doctors balance maintaining epistemic authority while also meeting public expectations. Second, in relation to self, they navigate the tension between professional role and personal exposure. Third, in relation to markets, they try to sustain their expert impartiality while using promotional tactics. Figure 1 summarizes these three dimensions of boundary work. The societal dynamics behind them are simultaneously at play, and, thus, the forms of boundary work overlap.

**Figure 1.**
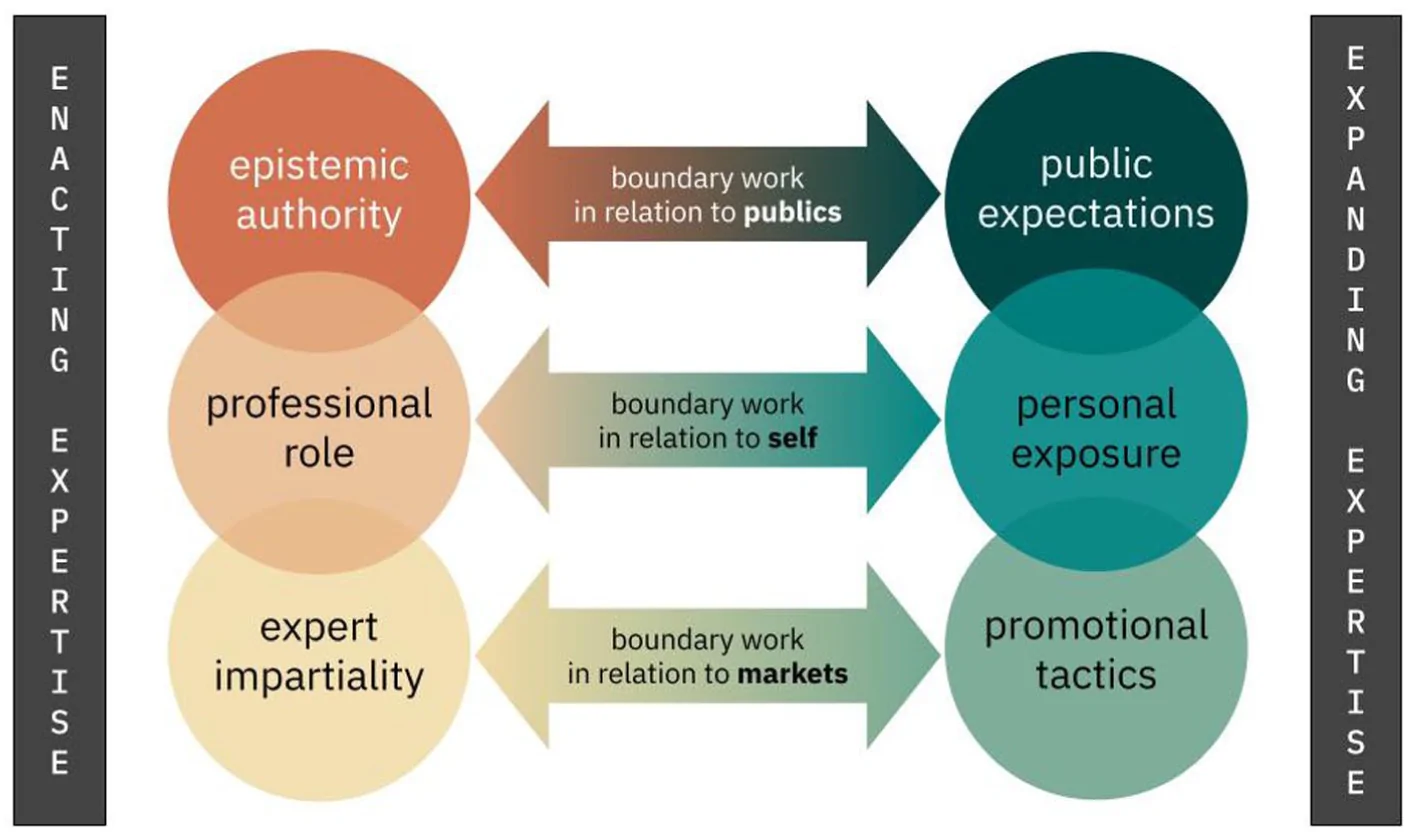
Types of boundary work by experts in the new media.

Reflecting on our research question, our empirical analysis looks into how doctors on Instagram, on one hand, enact expertise—more specifically, how they express epistemic authority, bring forward their professional role, and highlight their impartiality—and how they, on the other hand, expand expertise—concretely, how they cater to public expectations, share personal exposure, and use promotional tactics. To analyze how these elements are visible in the data, we utilize the concept of (self-)disclosure. Annie Li Zhang and Hang Lu (2023) researched scientists as influencers and noted that while professional self-disclosure improves their credibility, other strategies might help them reach wider audiences. Zhang and Lu (2023: 3) defined professional self-disclosure as the “sharing of either professional experiences and research related to a scientist’s career or blog and podcast-related content related to an influencer’s interests” and personal self-disclosure as “the sharing of personal interests, hobbies, and other non-science related information since such information is unrelated to a source’s scientific experiences.” In addition to these two dimensions, however, we observed a notable amount of content promoting a third party’s products, services, or ideas. Therefore, we added a third category: promotional disclosure.

## 3. Methodology

### Data

To analyze Finnish medical doctors on Instagram, we created a list of doctors active on the platform. The list of accounts was compiled by searching Instagram with relevant keywords (such as “medical doctor,” “gynecologist,” and “surgeon”), searching news outlets for mentions of doctors active on social media, and reviewing accounts that other doctors or professional organizations followed on Instagram. Based on this search, we listed doctors who have a medical license and an open Instagram account, on which they indicated their profession. The indication of the profession was visible either in their user name (e.g. using “dr” or “doctor” as a prefix or suffix to one’s first name), profile picture (a picture of oneself in a doctor’s coat), account description (e.g. listing one’s medical degree and area of specialization), and/or at least in 1 of their 10 most recent posts (posting about health or medicine or one’s work). Most of the listed doctors indicated their profession in all of these ways, but those who did not and used Instagram only for private purposes, were removed from the list. The search produced 60 individuals, and we focused on the 20 with the most followers in April 2022. We cross-referenced their right to practice medicine with the National Supervisory Authority for Welfare and Health (Valvira).

The data consisted of all Instagram posts from 20 doctors from the beginning of 2020 to the end of 2021 (*n* = 2125), and the analysis accounted for audiovisual, visual, and textual elements in both the post and its caption. The data do not contain Instagram Stories or Reels. The posts were collected using CrowdTangle, a service previously provided by Meta for researchers to collect and analyze public social media data. Individual doctors had published 4–320 posts, averaging 107. The average number of likes per post was 893, and the average number of comments was 28. When the data were collected, follower counts ranged from 1400 to 47,000. The doctors’ ages ranged from 28 to 53, and most of them were female (85%). Some worked in the public sector, and some in the private sector; seven were specialists, and one was a dentist. Medical specialties included geriatrics, dermatology and allergology, surgery, sports medicine, gynecology and obstetrics, plastic surgery, and general medicine.

### Analysis method and process

Our analytical approach followed an abductive logic, which suggests allowing theory and empirical observations to amplify one another during the research process, and leaves space for hunches, explanatory propositions, and doubt (Tavory and Timmermans, 2022). Our data analysis proceeded in three phases, combining qualitative content analysis with theory-driven close reading. To analyze doctors’ enactment and expansion of expertise, we first examined the different forms of (self-)disclosure (see Table 1).

**Table 1.** The final coding scheme.

Professional	Content related to doctors’ work or studies, medical content, health education, and/or content showing doctor’s garments or equipment.
Personal	Content related to one’s person; content related to one’s personal experiences, views, or emotions; content related to one’s free time or other engagements, and/or content showing a recognizable photo of oneself.
Promotional	Content related to promoting an identifiable third party to sell something or encourage the use of specified services (public or private, for- or non-profit, must include a call-to-action or a sponsor) or promoting one’s products or services (other than one’s Instagram account).

First, each author acquainted themselves with a randomized subset of 100 posts and wrote down observations related to the personal and professional self-disclosure used in those posts. Then, the notions were discussed, and a coding scheme was developed to classify the whole dataset. As expected, both personal content (e.g. sharing one’s everyday life, feelings, thoughts, and selfies) and professional content (e.g. fact sheets, presenting doctors’ work) were heavily present in the data, often combined in a single post. Based on our observations, we added the third type of disclosure, promotional disclosure. This first round of analysis also guided our theoretical orientation. First, after this round we returned to our theoretical starting point of expansion of expertise and followed the hunch that the interesting things happen when these types of self-disclosure overlap, thus, when boundary work takes place. Consequently and abductively, we allowed this empirical work to frame the three dimensions explained in theoretical background: relations to the publics, the self, and markets.

In the second phase of the analysis, we developed and tested the coding scheme by all four authors using randomized subsets of 50 posts. After each round, we calculated the intercoder reliability, and the label definitions were revised to resolve discrepancies. After three rounds of coding, intercoder reliability of α = .873 was reached, thus exceeding the commonly required α > .800 (Krippendorff, 2004). The entire dataset (*n* = 2125) was then labeled per the final coding scheme (see Table 1), with each author labeling one-fourth of the data. In addition, we also marked the topical focus of each post to get an overview of the topics present in the data.

In the third and final phase of the analysis, we focused on our primary interest: boundary work. To do this, we conducted a qualitative analysis of posts in which the code of professional content overlapped with either personal or promotional content (*n* = 956). In Figure 2, these overlaps are circled with a dashed line. Our analysis guideline was to categorize the different types of boundary work introduced in Figure 1: how doctors balance their epistemic authority and public expectations, professional role and personal exposure, and expert impartiality and promotional tactics. Again, observations were first written down by all authors working with a set of the data, after which we held several peer debriefing sessions (Lincoln and Guba, 1985) to collect our observations.

**Figure 2.**
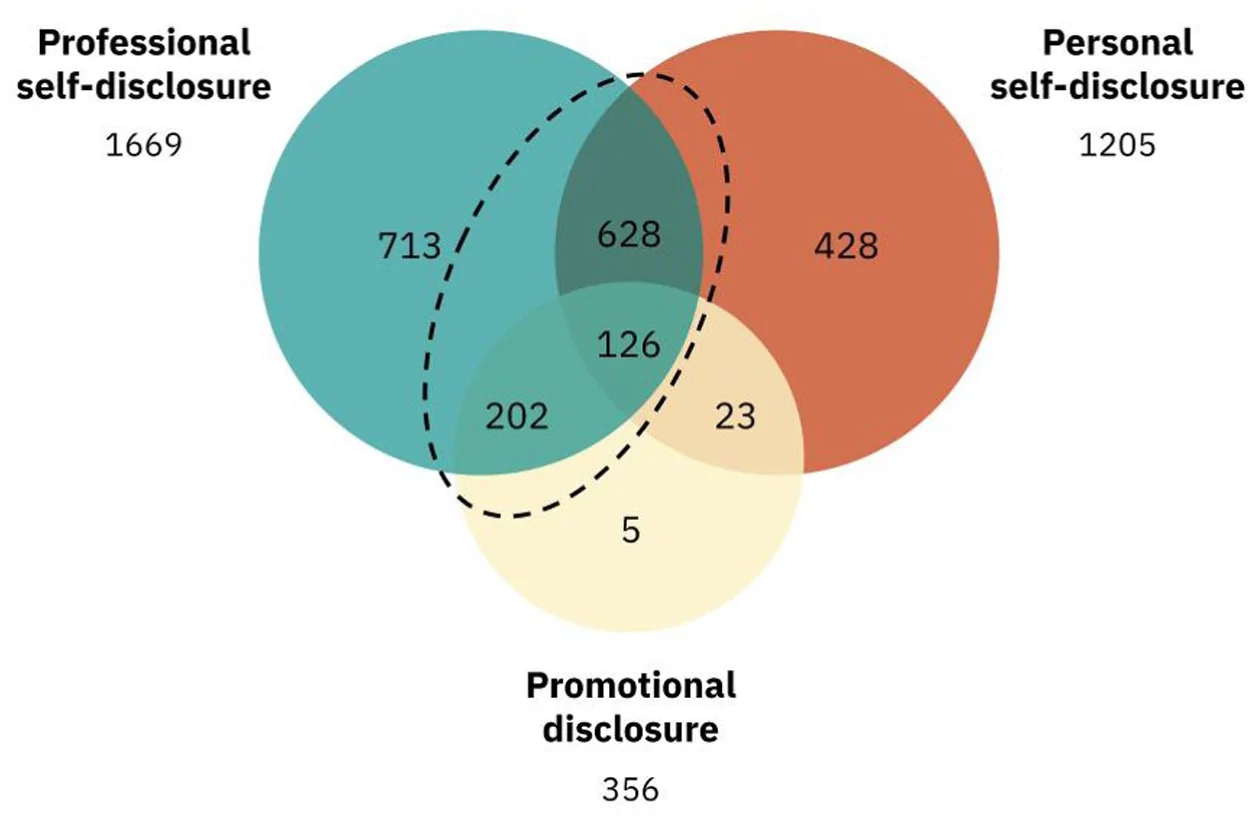
A Venn diagram overview of the data, including personal, professional, and promotional (self-)disclosure and their overlaps. The dashed line marks the qualitatively analyzed data.

Next, based on the close reading of the data, we describe the key aspects of the findings and discuss the boundary work practiced by the studied doctors on Instagram. Excerpts from the data are marked with an identifier of the doctor who published the post (A–T) and a chronological number in the data (1–2125).

## 4. Findings

*Professional* content was plentiful as all the studied accounts were tied to the account owner’s profession—most indicated their profession or medical degree in their user name and/or in the account description, but also in their posts by explicitly discussing health topics and stressing their professional take on them. Many posts resembled public service announcements or social marketing campaigns in that they focused on a specific health topic or question (e.g. “Endometriosis and psychological symptoms,” M986; “Does whey protein cause acne?” Q787; instructions for using face masks, D322, J824, O1513, E1604, L1607, L1902, H1915). While most professional content was directed at the general public, there was also content produced for peers for educational or collegial purposes (e.g. “How to remove sterile surgical gloves,” R1791). The professional content was typically created using a correct tone of voice and language. Still, Instagram-friendly visuals, little humorous remarks, and emojis were often used to make the content attractive and easy to digest.

*Personal* posts focused on facial images, including selfies, of the doctors. Being individuals and not organizational actors, many doctors wanted to share their perspectives and persona alongside the professional knowledge they shared. While some posted personal content in addition to professional content, as if speaking to a friend (e.g. family photos, holiday plans), others kept the account entirely professional.

Four accounts produced most of the *promotional* content in the data. Some advertisements for health-related products were present, and some promoted medical practices or services. However, most promotional content went beyond doctors’ immediate profession and included advertisements for clothing brands, food products, or cosmetics. Some promotional posts did not contain a financial incentive, such as advertising charitable causes.

Next, we will introduce our findings regarding the different dimensions of boundary work—in relation to publics, self, and markets. We have selected the most representative posts as examples of each type of boundary work.

### Boundary work in relation to publics

Boundary work in relation to publics is marked by maintaining epistemic authority while navigating the public expectations that active content production on Instagram makes evident. There was some variation in who doctors considered their primary audience; some target laypeople, some colleagues, and some those applying to medical school.

Doctors are intentionally creating content for an audience. They have public Instagram accounts, and they often address their followers directly (“I hope you knew that . . .”; “What if you . . .”; “What’s your take on . . .”). Doctors use various strategies to engage their followers, like writing thought-provoking questions and encouraging comments. While imagery and language made it clear that doctors responded to general expectations for Instagram content, the message was often medically focused. Both humorous images and infographics were present in the material, aiming to attract and educate audiences. In the example post below, a doctor discusses work-related well-being in the healthcare sector using a humorous superhero photo and encourages audiences to share their views on the topic:

[A drawing of a comic book hero with a text “Heroes, are there any?”] Coping at work has increasingly become a topic of discussion lately. The effects of COVID-19 on healthcare professionals were somewhat paradoxical last spring, as the number of patients suddenly decreased, and the workload for many doctors and nurses became more manageable. (. . .) For those working in demanding positions, it is essential that the workload is manageable and meaningful – at times even lighter, so that peak loads can be handled better. (. . .) What do you consider an important method for supporting employee well-being? #workwellbeing #medicaleducation #generalmedicine #nomoresaltmines (F1144)

The boundary work in relation to publics was characterized by doctors keeping the medical input in their own hands but allowing audiences to participate in defining the agenda. In the posts, doctors acknowledged their followers and kept addressing questions and topics audiences suggested or doctors had encountered in real-life encounters. The relationship built with the audience often had a friendly, everyday, and understanding tone, but the flow of information was one-directional. In the example below, a doctor explains why gynecologists ask their patients certain questions and encourages audiences to comment if they come up with other reasons:

[A selfie of a doctor with a mask on using an ultrasound machine (Figure 3)] When was your last period? You’ll be asked this almost every time at a gynecologist’s appointment
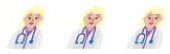
 What information do we get from this? 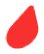 What week of pregnancy might you be in? 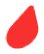 How large of a pregnancy can I expect to see on an ultrasound? 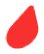 Is the uterine lining thick, triple-layered, or thin (as seen on ultrasound)? 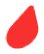 Is the abdominal pain due to ovulation? A cyst? Bleeding in the abdomen? Endometriosis? 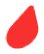 Is the bleeding spotting or your period? 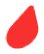 Could the psychological symptoms be PMS/PMDD-related? (. . .) Can you think of anything else that can be deduced based on the cycle? 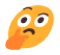 (M714)

**Figure 3.**
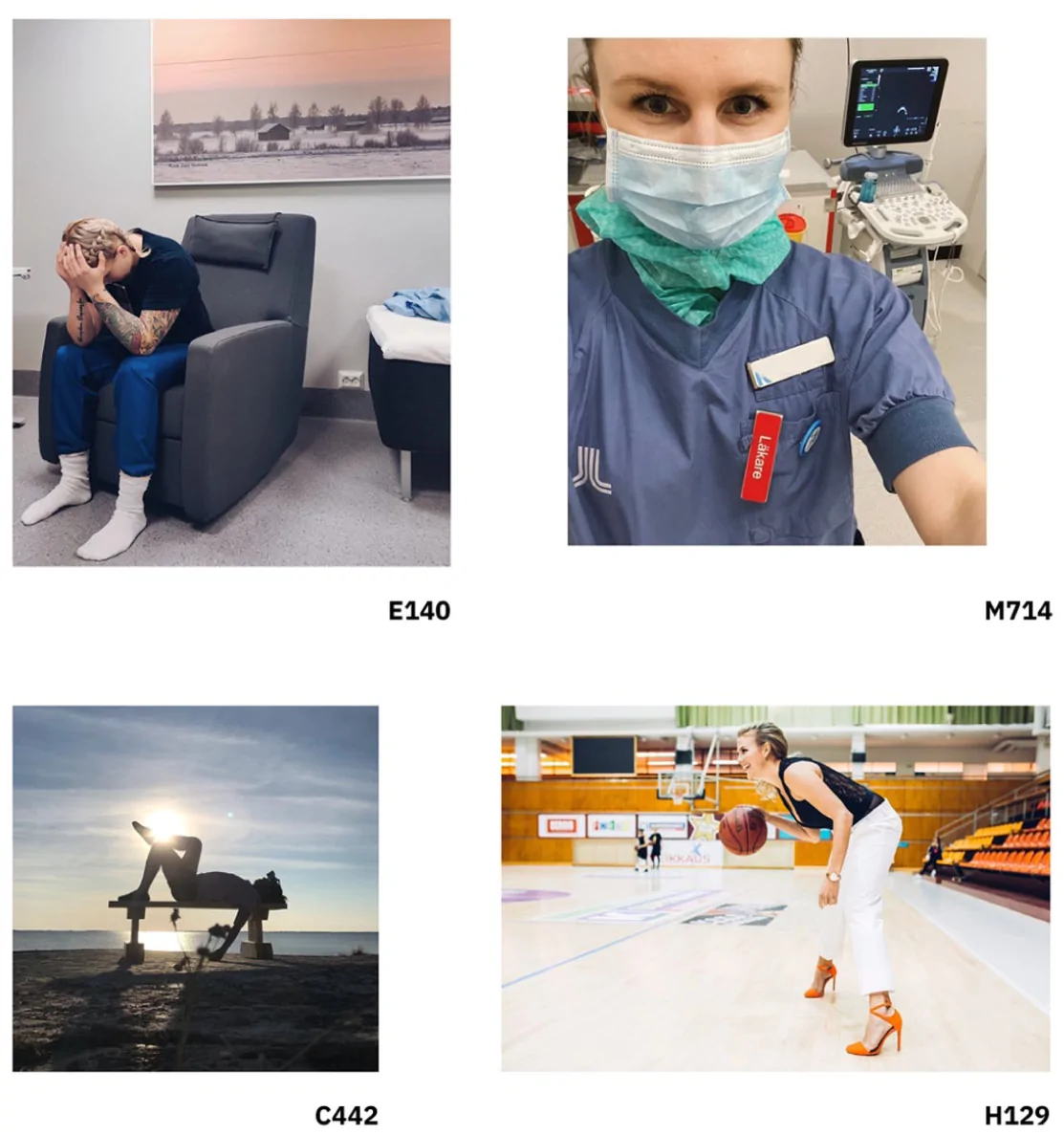
Images drawn from the research data and used as representative examples in the findings. *Note*: The images are used with permission of account owners.

Epistemic authority was often shared with colleagues—doctors and nurses. Doctors expressed trust in science and often mentioned research institutions and journal articles as sources for the information they shared in their posts. In a few cases, epistemic authority was extended to practitioners outside medicine, like yoga instructors. Typically, however, the doctors criticized health and well-being influencers (directly and indirectly), especially those who spread mistrust toward healthcare, biomedicine, and medical professionals. This way, they highlighted their own and their profession’s expertise compared with self-taught experts or influencers, making it known that the latter might be misleading publics. In the data, there were many posts that discussed how social media can accelerate the spread of misinformation and unrealistic expectations. In many ways, influencer responsibility, especially related to misinformation, was raised. The doctors criticized complementary and alternative medicine supporters and practitioners for censoring medical professionals’ comments on their posts, tried to prevent the spread of misinformation by closing comment sections of their (mostly COVID-19-related) posts, and discouraged their audiences from giving attention to misinformation, if even to refute it. This way, doctors tried to educate their followers to be media literate, strengthening the boundary between their science-based expertise and attention-based social media:

[A smiling selfie in everyday clothing] Let’s hijack hashtags (. . .) to share beneficial information instead of untruthful. (. . .) JUSTIFY YOUR BEHAVIOR IN EXCEPTIONAL TIMES FOR EXPERT RECOMMENDATIONS, NOT GUESSWORK ON SOCIAL MEDIA!! (. . .) (E1375)

[A screenshot of a tweet by another social media user urging people not to share misinformation] You see something on social media that upsets you. It might be a political statement, a text violating human rights, or a faulty, over-the-top health-related claim. (. . .) 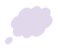
What you should NOT do?⁣ 
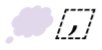
Take a screenshot or copy-paste, share, or even tag this source of nonsense with a caption “bullsh*it” or whatever. (. . .) By sharing untrue or offensive content, you are actually spreading misinformation. (. . .) 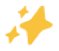 This is why I did not bring up or correct the faulty health claims you send via dm’s to me. (. . .) (L1742)

A clear boundary was drawn when individual followers had sent direct messages with personal medical information. Doctors commented that they could not answer these direct messages for legal or ethical reasons (“I get a lot of questions (. . .) and even though it would be wonderful to reply to all of them, in most cases, it’s not legally possible (. . .) for information security reasons,” P1474).

Doctors also mapped out the boundaries of their professional world. There were instances when doctors acknowledged the limits of medical knowledge, the challenges posed by limited resources, and the room for human error, which can help explain and justify why the care received is not always what the patient hoped for. This way, they both recognized the limitations of their profession and explained why even their expertise cannot offer solutions in all instances. It also differentiates them from actors who do not recognize the possibility for error.

To summarize, doctors addressed and engaged publics in several ways but confined the epistemic authority in health questions to traditional scientific-vocationally trained professionals.

### Boundary work in relation to self

The boundary work between professional role and personal experience was characterized by content that expressed approachability but still foregrounded medical work, education, and knowledge. Stereotypically cold, distant, fact-oriented experts were mainly absent. Instead, various selfies in doctors’ scrubs and coats were attached to posts that discussed work, politics, free time, and family recollections.

The most common example was sharing a casual selfie from work, describing the workday, and offering a “behind the scenes” glimpse into a doctor’s job. Doctors represented multiple aspects of their life while emphasizing their professional role. However, in some posts, doctors took an entirely different approach, mainly sharing personal or promotional content and using their profession as a reassuring point of interest. The examples below reflect these approaches:

[A selfie of a doctor in scrubs and a mask on in healthcare center surroundings] Visiting the public sector for skin cancer surgery. Had to experience [infamous IT system], of course! 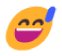

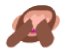
 #extremesports 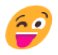 #doctor #doctorsofinstagram #dermatologist #dermatosurgery #doctorsofinstagram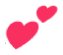 (Q753)

[A person lying on a bench in the sun (Figure 3)] In vacation mode. In my current life situation, I’m definitely not lounging by pools or enjoying long stretches of time in these kinds of positions and moments. But I always try, whenever possible, to take a relaxed attitude, put my feet up, and rise above the daily grind, even if it’s just with a good cup of coffee – sometimes even with a glass of wine. Intensive work and small children don’t make it easy to take time for laziness. But even a small moment is enough! Laziness in the right moments keeps the mind together 
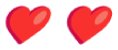
 #doctorlife #mom #gynecology #vacationmode #lazinesswithgoodconscience (C442)

In the posts, doctors’ emotions toward their work were present. They expressed, for example, sadness and anxiety in the worsening COVID-19 pandemic, shared gratitude for being able to help people, and reflected on what the death of a patient feels like or how they fear making mistakes, as those have severe consequences. The apparent self-reflection was directed to the reader, using eye-witness position and firsthand professional experience to highlight their credibility as experts. While anecdotal examples and emotional pleas are not that valued in science-based discussions, they seem to be a popular way to increase approachability in surroundings such as Instagram. In the example below, a doctor discusses her feelings and firsthand experiences of treating COVID-19 patients and expresses an emotional plea for people to get vaccinated:

[A doctor in scrubs, sitting in a chair with head laid on hands (Figure 3)] At this moment, treating corona patients feels wrong, and the feeling is so sad. (. . .) It seems too big of a lesson to lie in an intensive care unit deeply anesthetized in a respiratory machine just because one has declined the corona vaccine (. . .). I feel for those small children who do not even understand (and maybe better so) what it means when their parent has ended up in intensive care (. . .). Not to even mention the “get well soon” cards hanging over the bed, made by hopeful children. (. . .) The vaccination is not mandatory, but in the current situation, the choices are pretty much getting the corona disease or the vaccine. (. . .) Do you want to be a parent to your children in the future and stay alive? Or gamble and see what cards you get? (. . .) (E140)

Besides sharing their experiences as medical professionals, the posts also covered doctors’ perspectives as patients and their personal health-related decisions. The intimacy of shared information ranged from general content like exercise photos and nature walks for mental health to deeply personal stories about pregnancy, childbirth, autoimmune disease experiences, as well as mental health diagnoses and coping strategies. These personal narratives were consistently woven into broader discussions about the healthcare system, mental well-being, and illness treatment. Especially in the case of COVID-19 vaccines, doctors widely shared how they had taken the vaccine and encouraged audiences to do the same, accompanied by scientific arguments, as in the example below. The use of personal experiences and examples is a common tactic among self-taught experts in questions of health (Huovila and Saikkonen, 2016; Jauho, 2016), and previously, scientific experts’ own vaccination decisions have been a topic of interest for journalistic media (Boyce, 2006):

[An image of a smiling person with a plaster on their upper arm] The second dose of the corona vaccine received 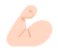

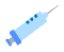
 (. . .) I got the Pfizer-Biontech vaccine that includes coronavirus’s messenger-RNA (mRNA). Using it, the body will code the virus’s surface protein, against which immunity will develop. (. . .) 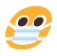 I experienced no side effects besides arm pain that lasted for a day. (. . .) 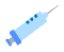 Studies suggest that a week after the second dose the protective efficacy is already 95%. (. . .) I hope those considering getting vaccinated can access reliable information instead of disinformation. (. . .) I have a strong trust in science 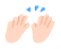

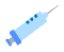
 Source: [Finnish Institute for Health and Welfare] #medicalschool #doctor #coronavaccine (K1178)

Some doctors raised political topics, either advertising their candidacy in local elections or expressing advocacy in health-related themes. Examples of the latter include supporting women’s rights, endorsing vaccinations, defending the universal healthcare system, and the right to safe abortions. While these themes are highly debated in some countries, such statements are not provocative in the Finnish context. Doctors primarily advocated from their professional perspective, suggesting they viewed these political statements as integral to their work. In the example below, a doctor argues for the right to safe abortions, as they are low-risk and increase public health:

[A doctor in scrubs with one fist lifted in air] Today is International Safe Abortion Day. 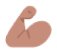 (. . .) Abortion is a woman’s own business. If she feels it as an existential question, it is one. If it feels physically or psychologically hard, then it is that. If a woman does not experience abortion as an existential question, then it is not one. 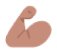 (. . .) Abortions (safe and legal abortions) do not cause many medical problems. Most abortions are done with pills at home. (. . .) 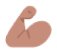 Much research has shown that safe and legal abortions increase public health. Why, then, shouldn’t women have the freedom to make choices about their own bodies? (M260)

Part of this boundary work was doctors’ self-reflection of their media presence in social and legacy media. In these posts, doctors wondered if they expressed themselves enough without simplifying information, discussed their reasons for being on social media, and justified their choices. This open self-reflection promoted their media presence, but, on the contrary, set it up against the professional standards and epistemic authority that doctors generally hold. Some had the explicit intention to “humanize” the public image of doctors, as in the example below, suggesting that doctors can sometimes feel distant and hard to approach. In the example, a doctor shares a photo of themselves in short sleeves, revealing their tattoos, and discusses how creating only science-based content would make social media work feel tedious:

[A person sitting at a bus stop in shorts and a t-shirt, showing tattooed arms] What kind of content is it appropriate to publish as a doctor? 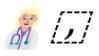 (. . .) Should a doctor only publish professional content on social media? (. . .) One of my goals on social media is to humanize the general image of doctors. Of course producing trustworthy knowledge, too, but if my account was only that, it would be much duller and, well, humaneness would be far off. And I wouldn’t have the energy to do this if the content was strictly science. (. . .) (E1629)

In many ways, doctors on Instagram are more visible as opinionated, “whole” people with feelings and thoughts than as detached, cold professionals. They use their experiences as doctors *and* patients to increase their credibility and invoke interest and affective reactions in their audiences. Like other influencers, they build relationships with followers through their posts and pursue authenticity (see Munnukka et al., 2019; Pöyry et al., 2019).

### Boundary work in relation to markets

As members of a profession with strict ethical norms and regulatory control, doctors in our data negotiated the boundary between their expert impartiality and the different promotional tactics made possible by the platform. There were stark differences in how this was done: a few doctors had clearly more visible commercial aims and collaborations, and a few more used promotional tactics without clear commercial benefits.

Expert impartiality was at stake in three different ways. First, doctors offered speaker gigs, online courses, podcasts, and books, which resembled the content and products created by influencers. Wellness products and content, in most cases, do not break any of the doctors’ traditional professional norms, making them a more manageable and ethically suitable approach to monetizing Instagram attention compared with medical services and products. This was also ensured through disclaimers: for example, when selling their online course on iron deficiency (P1513), a doctor reminded how various things other than iron deficiency can cause fatigue. Using their online persona as a platform, they were expanding to new areas of expertise while still utilizing their professional knowledge:

[A selfie of two people in a podcast studio] 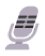 In exactly a week from now our [podcast name] first episode will be published and I AM BURSTING WITH EXCITEMENT! 
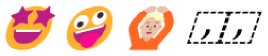
(. . .) Episode themes in the future will include body image, trouble sleeping, antibiotics, psychiatry of the future. . . (. . .) 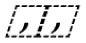 Press 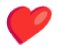 if you’re planning to listen 
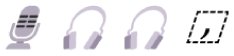
 (. . .) #doctor #medicine #medschool #health #wellbeing #podcast #podcasts (L2122)

Second, doctors also used their Instagram fame to sell various kinds of products and services offered by others, utilizing their credibility as medical experts on the matter in question. In these cases, the boundary work was visible in disclaimers—for example, how a smartwatch is not a medical device but a way to support one’s well-being (A994). In the example below, a doctor does a commercial collaboration to promote a device to treat urinary incontinence but adds a disclaimer that the treatment should always be done with the help of a trained physiotherapist:

[A person standing on a rock on the beach, dress flowing in the wind] Commercial collaboration with [company name]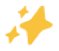 Urinary incontinence is a very common condition that affects all age groups. (. . .) The causes of incontinence are complex, and often the issue stems from dysfunction in the coordinated action of the core muscles, diaphragm, and pelvic floor. (. . .) [Brand name] is the world’s first patented solution for urinary incontinence and is easy and invisible to use. (. . .) However, the cause of urinary incontinence should always be determined and treated with the help of a skilled pelvic floor physiotherapist. (. . .) #pelvicfloor #urinaryincontinence #women #incontinence #[brandname] #[companyname] #womenshealth (H239)

Third, in some posts, the focus was lifestyle-oriented. In these cases, the references to the medical profession or scientific vocabulary were used more liberally and about matters unrelated to a doctor’s profession or education. Typical topics were holistic health and wellness. In the example below, a doctor discusses “love hormones” and their meaning during and after an athlete’s career. This dimension highlights the blurring of boundaries between traditional experts and influencers:

[A person playing basketball in high heels (Figure 3)] I was a guest on the @taggedaccount podcast, and we talked about athletes’ career transitions and love hormones. At its best, sports, especially team sports, provide us with a steady stream of love hormones. Shared goals and working towards them ensure a constant flow of dopamine. (. . .) When a career ends, all of this can disappear at once, and the hormone balance that promotes well-being must largely be rebuilt. (. . .) We also talk about why most athletes break up after their careers end, and how the @taggedaccount community influenced my own growth and healing journey. #embracelove #sports #careertransition #lovehormones #love #basketball #icehockey (H129)

Balancing a doctor’s profession and social media promotion has practical implications: being notable on social media requires a lot of time and other resources. Doctors also reacted to content from other influencers and participated in ongoing social media debates and challenges. Even those doctors who mainly focused on scientific and professional content used their time and other resources to gain visibility and fit in on the platform, posting regularly and learning new skills, like video editing.

Surprisingly, direct advertising of doctors’ own practice or medical services was almost nonexistent, and most refrained from paid advertisements and commercial collaborations altogether. From the totality of posts with professional self-disclosure, one-fifth included some promotional disclosure, and only a few accounts were responsible for most of it. This suggests that promotionally oriented content for most doctors on Instagram is a minor task in terms of monetization, and the true motivations for courses, podcasts, and books lie elsewhere. On the contrary, the promotional content was heaviest on a few accounts, suggesting that social media had become a relevant source of income for some.

## 5. Discussion

In this study, we explored the enactment and expansion of expertise on Instagram. Previous research on expanding expertise, especially in health, has focused on how traditional expertise is challenged (Gunnarsson and Elam, 2012; Huovila and Saikkonen, 2016; Jauho, 2016; Kata, 2012) and how citizen stakeholders have fought to have their lay expertise recognized (Epstein, 1996). We contribute to existing research by turning the spotlight on traditional professionals, medical doctors, and how they enact and expand expertise.

To analyze this enactment and expansion, we mobilized the concept of boundary work (Gieryn, 1983). We theorized that experts in social media are required to do boundary work regarding publics, self, and markets. This boundary work concurrently enacts and expands expertise. The results of our analysis, which investigated doctors’ boundary work on Instagram, are summarized in Table 2.

**Table 2.** A summary of the results.

Boundary work	Underlying development	Enacting expertise	Expanding expertise
In relation to publics *Between epistemic authority and public expectations*	The public assessment and challenging of traditional epistemic authorities	Taking epistemic authority over publics	Including audiences in setting the agenda Building parasocial relationships
In relation to self *Between professional role and personal exposure*	The personified tactics used by novel experts	Medical science, work, and education as the main content topics	More humane, approachable representation of doctors through sharing personal imagery, emotions, and experiences Political advocacy
In relation to markets *Between expert impartiality and promotional tactics*	The rise of promotional culture	Disclaimers and professional norms Lack of advertising of medical devices or one’s practice	Creating and selling online courses, books, and podcasts Commercial collaborations and influencer marketing

A large portion of doctors’ boundary work enacted expertise. In relation to publics, doctors expressed their own and their profession’s epistemic authority over laypeople and self-trained experts. In relation to self, their professional role was communicated through visual imagery and discussions of workplace and medical practices. In relation to markets, doctors protected their professional impartiality by adhering to their professional norms. They advertised very little of their own medical practice or specialty.

However, doctors also expanded expertise. In relation to publics, doctors took note of public expectations and addressed the worries and questions of their followers, aiming to connect with them. In relation to self, the expansion of expertise was most clearly visible, as doctors shared personal information and expressed emotions toward many issues. They not only relied on their professional expertise but also on personal exposure, as many self-trained and alternative experts do (Deml et al., 2019; Huovila and Saikkonen, 2016). In relation to markets, doctors adopted new earning models, such as commercial collaborations, books, and online courses. These more lifestyle-oriented monetization models resemble how lifestyle experts combine expertise with consumership, associating desirable lifestyles with the purchase of certain products (Lewis, 2010). In most cases, the commercial collaborations had no connection to their professional expertise.

We also contribute to the research on how epistemic authority and recognition are sought in different media (Christensen, 2016; Egher, 2020; Huovila and Saikkonen, 2016; Lewis, 2010; Setälä and Väliverronen, 2014; Shanahan, 2010; Zhang and Lu, 2023). These studies typically focus on new types of public experts, who are seeking audiences for their claims to expertise. Medical doctors are not addressed from this perspective, because traditional professionals are the norm everyone else is compared with. However, in the new media environment and due to the changes in the social conditions of expertise, some doctors find they have to enter the public realm and gain the attention of the audiences.

Doctors mobilize the resources offered by Instagram as a platform, mainly to spread medical knowledge, but by adapting to it, their content starts to resemble general Instagram content. While it is tempting to say that the boundaries between expertise and non-expertise are blurring on social media because they look the same, the problem is, as Peter Weingart (2012: 24) has stated regarding the boundaries of mass media and science, “they still have to rely on the very distinctions which are supposedly disappearing.” Many doctors gain followers on Instagram precisely because they are professional experts and can thus differentiate themselves from the mass of accounts. Based on our results, doctors use this opportunity to create distance between themselves and self-made experts, making their profession known and explaining how their professional expertise has developed over time.

Our data give the impression that doctors approach their media work systematically and reflect on their choices. However, creating well-planned content takes time and resources. As scientific institutions face increasing criticism and healthcare systems worldwide struggle with growing efficiency demands, social media can help doctors defend their stance and the scientific approaches they are committed to—but it can also become yet another area of work that doctors are expected to manage.

When examining the expansion of expertise from a normative perspective, it is tempting to value it negatively and, thus, to view traditional experts’ actions to expand expertise as alarming. However, our data suggest that such expansion generates increased interaction with followers, more personified representations of experts, narratives that help to distribute information, and new tools to share expert knowledge with audiences. While there is a risk that some audience members can no longer distinguish between a self-taught influencer and a doctor, a great deal of expert information would not have reached audiences without doctors taking on social media.

### Limitations and future research

The study has limitations that relate to the nature of our research data. First, we focused only on Instagram, a platform dominated by specific types of content and user profiles. Analyzing Twitter or TikTok could have produced different kinds of results. Despite our efforts to compile a list of the most-followed medical doctors, it may be incomplete. Second, we analyzed only Instagram Posts, not Instagram Stories or Reels. Stories are visible only for 24 hours. Users might publish more spontaneous or personal content to Stories than to Posts, which are, by default, more lasting (Kreling et al., 2022). Third, the data were collected during the COVID-19 pandemic. As it was an exceptional time, doctors might have been more active than usual, and they might have had different motivations for active boundary work than usual.

We focused on the most-followed Finnish doctors on Instagram to understand the communication styles of the most influential individuals on the platform. Consequently, the results do not reveal the actual distribution of personal, professional, and promotional posts among all doctors. Moreover, we don’t know which posts were most popular among the followers. Some posts receive more visibility than others, but the view count data were unavailable. In addition, the structure of the Finnish healthcare system influences the results. In 2022, among working-age doctors, 16% were employed by private operators and 11% worked as private entrepreneurs (Lääkäriliitto, 2023). However, many doctors hold dual roles, working in both private practices and public health centers (Lääkäriliitto, 2023). Because of this, Finnish doctors likely have fewer financial incentives for self-promotion or client acquisition.

Future research could investigate how followers of medical doctors perceive different types of social media content and its potential effects on them. Another future research avenue includes studying social media boundary work in other fields of expertise or different national or cultural contexts.

In our study, we focused on public expertise of a well-known profession, whose services are familiar to the audience. Previous research suggests that personal self-disclosure can negatively affect expert credibility (Rödder et al., 2025; Zhang and Lu, 2023), but in those studies, the experts were researchers without direct contact with the public. Thus, the boundary work of less visible experts might take different forms compared with doctors or other well-known professions audiences have personal experience with.
